# AutoMeDIP-seq: A high-throughput, whole genome, DNA methylation assay

**DOI:** 10.1016/j.ymeth.2010.04.003

**Published:** 2010-11

**Authors:** Lee M. Butcher, Stephan Beck

**Affiliations:** UCL Cancer Institute, Paul O’Gorman Building, University College London, 72 Huntley Street, London WC1E 6BT, UK

**Keywords:** DNA methylation, Automation, Whole genome, High-throughput sequencing, MeDIP

## Abstract

DNA methylation is an epigenetic mark linking DNA sequence and transcription regulation, and therefore plays an important role in phenotypic plasticity. The ideal whole genome methylation (methylome) assay should be accurate, affordable, high-throughput and agnostic with respect to genomic features. To this end, the methylated DNA immunoprecipitation (MeDIP) assay provides a good balance of these criteria. In this *Methods* paper, we present AutoMeDIP-seq, a technique that combines an automated MeDIP protocol with library preparation steps for subsequent second-generation sequencing. We assessed recovery of DNA sequences covering a range of CpG densities using *in vitro* methylated λ-DNA fragments (and their unmethylated counterparts) spiked-in against a background of human genomic DNA. We show that AutoMeDIP is more reliable than manual protocols, shows a linear recovery profile of fragments related to CpG density (*R*^2^ = 0.86), and that it is highly specific (>99%). AutoMeDIP-seq offers a competitive approach to high-throughput methylome analysis of medium to large cohorts.

## 1 Introduction

DNA methylation is an indispensible, tissue-specific epigenetic mark occurring predominantly at CG dinucleotides (CpGs) in mammalian genomes. It refers to the post-replication maintenance or *de novo* addition of a methyl group to the carbon-5 position of the cytosine pyrimidine ring by DNA methyltransferases to form 5-methylcytosine (5mC). DNA methylation has important consequences for gene expression by contributing to the remodelling of chromatin via recruitment of methyl-CpG-binding domain (MBD) protein complexes and subsequent chromatin modifiers. Disease phenotypes have been shown to arise when these activities are perturbed, resulting in undesired gene expression or silencing; this is most notable in carcinogenesis (for reviews, see [1,2]).

Although DNA methylation was discovered 60 years ago – and has been shown to be involved in human disease some 25 years ago – efficient technologies for genome-wide methylation analysis have only become available fairly recently with the introduction of the microarray and, more recently, second-generation sequencing, exemplified by Solexa (Illumina), SOLiD (Applied Biosystems) and PSQ (Roche) chemistries. To date, most studies have focussed on DNA methylation at gene promoters and CpG islands, as methylation at these regions defines cellular identity and aberrant methylation here is likely to contribute rare but significant changes to the phenotype, however; recent evidence suggests that regions most likely to undergo change in DNA methylation may occur at loci outside gene promoters and CpG islands – so called CpG island shores [3]. Changes here are thought to be more frequent but also more subtle, thus contributing synergistically to disease phenotypes.

From a methodological point of view, assaying these putatively functional regions presents two issues. Firstly, analyses need to encompass the whole genome: there are >28 million CpG sites scattered throughout the haploid human genome (the methylome) and it remains technically challenging to capture the majority of these (for an overview of these methodologies, see [4,5]). Secondly, these techniques need to be scalable for use with large cohorts to maximise statistical power to elucidate reliable differentially methylated regions (DMRs).

Second-generation sequencing has eased the former burden; for example, Solexa sequencing coupled with bisulfite conversion (MethylC-seq) recently provided the first human DNA methylome at single CpG resolution [6]. At the current cost however, MethylC-seq is precluded from routine use on large cohorts and leaves the latter burden unresolved.

One approach that strikes a good balance between coverage, resolution, throughput and cost is to combine second-generation sequencing with the methylated DNA immunoprecipitation (MeDIP) assay [7]. MeDIP is an enrichment-based technique [8] that uses an antibody against 5mC to capture the methylated fraction of a genome suitable for array-based analysis (MeDIP-chip; [9–11]) or sequencing-based analysis (MeDIP-seq; [7]). The main advantage of MeDIP is that genomic capture is relatively unbiased and not limited to restriction sites or CpG islands. At ∼100 bp resolution, MeDIP-seq offers comparable coverage at much lower cost than MethylC-seq. However, throughput is currently limited due to the manual procedure of the MeDIP step, an issue we address here.

Traditionally, issues with throughput have been alleviated using automation – an approach used here. The recent development of the SX-8G IP-Star by Diagenode opens the way to perform up to 16 automated MeDIP (AutoMeDIP) or ChIP (AutoChIP) assays per run that, with some considerations, are compatible with subsequent second-generation sequencing. In this study, we focus on AutoMeDIP and describe a quality control (QC) procedure using *in vitro* methylated lambda (λ) DNA sequences to demonstrate reliability, sensitivity and specificity of the method. In addition, we provide practical considerations for combining AutoMeDIP with Illumina Genome Analyzer II (GAII) library preparation for subsequent sequencing (AutoMeDIP-seq). We believe AutoMeDIP-seq offers a competitive approach to high-throughput whole genome methylation (methylome) analysis of medium to large cohorts.

## 2 Methods

Although the focus of this Methods article is the application of AutoMeDIP, we begin by describing the generation of the λ-DNA fragments for the QC procedure. We next describe the automated platform itself and finally, a workflow.

### 2.1 Reagents and equipment

λ-DNA, restriction enzymes and methyltransferases (and their appropriate buffers) from New England Bioscience (Ipswich, MA). PCR buffers, Taq DNA polymerase and dNTPs from Abgene Ltd. (Epsom, UK). Primers and quantitative PCR master mixes from Eurogentec (Liège, Belgium). Zymo Clean & Concentrator-5 columns from Cambridge Bioscience (Cambridge, UK). QIAquick, MinElute and Gel Extraction kits, columns 96-well plate equivalents from QIAGEN (Crawley, UK). 50× Tris–acetate–EDTA from Thistle Scientific (Glasgow, UK). Low-melting-point agarose from VWR (Lutterworth, UK). MeDIP reagents, 8-strip tube magnetic rack, Bioruptor UCD-200 sonicator and SX-8G IP-Star automated MeDIP and ChIP platform from Diagenode (Liège, Belgium). Illumina Paired End DNA Sample Prep reagents from Illumina (San Diego, CA). BioAnalyzer 2100 and DNA chips from Agilent (Wokingham, UK). Genecatcher DNA purification kits from Invitrogen (Paisley, UK). ND-1000 spectrophotometer from NanoDrop (Wilmington, DE).

### 2.2 DNA

Lambda (λ) DNA was spiked-in against a fixed amount of sheared human genomic DNA. λ-DNA is an ideal spike because its genomic sequence shares no homology with human (or other mammalian) genomes, nor with Illumina GAII Paired End adapter, PCR primer or sequencing primer sequences.

#### 2.2.1 Generation of λ-DNA fragments

A total of six λ-DNA fragments were constructed using PCR. PCR was carried out in final reaction of 25 μl, containing 0.625 U Taq DNA polymerase; 1× reaction buffer containing 1.5 mM MgCl_2_; 0.2 mM each dNTPs; 500 mM each forward and reverse primers; and ∼6.6 × 10^−4^ pM (∼10,000 copies) λ-DNA. Reactions were incubated on a thermal cycler with the following conditions: (a) 94 °C for 2 min, (b) 32 cycles of 94 °C for 20 s, *T_A_* °C for 30 s, and 72 °C for 60 s, and (c) 72 °C for 5 min, where *T_A_* °C is the annealing temperature indicated in Table 1.

PCRs were purified on Clean & Concentrator columns and eluted in 10 μl molecular biology grade water. Fragments were size-evaluated using an Agilent 2100 Bioanalyzer with DNA 1000 chips. On average, a single PCR generates enough fragments for more than 500,000 spike-in experiments.

#### 2.2.2 *In vitro* methylation of λ-DNA fragments

Each purified fragment was split into two aliquots; fragments from one aliquot were *in vitro* methylated while fragments from the other were unprocessed, thereby forming unmethylated counterparts. *In vitro* methylation was accomplished by incubating λ-DNA in a final reaction volume of 20 μl containing 1 μg λ-DNA, 2 μl 10× NEBuffer 2, 4 U SssI methyltransferase and 160 μM *S*-adenosylmethionine (SAM) at 37 °C for 1 h then 65 °C for 20 min. DNA was purified on Clean & Concentrator columns and eluted in 20 μl dH_2_0.

To test the extent of *in vitro* methylation each amplicon was designed to contain at least one 5′-ACGT-3′ motif, which is recognised by the CpG methylation-sensitive restriction endonuclease, *HpyCH4*IV: cleavage at this motif only occurs in the absence of CpG methylation. *In vitro* methylated PCR fragments were incubated at 37 °C for 1 h, and stopped by heat inactivation at 65 °C for 20 min in a final reaction volume of 25 μl containing 1 μg λ-DNA, 2.5 μl 10× NEBuffer 1, and 10 U *HpyCH4*IV. Following incubation, λ-DNA was purified on Clean & Concentrator columns and eluted in 10 μl dH_2_O. Each purified λ-DNA product was evaluated for endonuclease activity using an Agilent 2100 Bioanalyzer with DNA 1000 chips; molarities of each fragment were quantified against a standard of known concentration using qPCR (using nested qPCR primers; see Table 1) to construct an equimolar spike-cocktail containing each *in vitro* methylated fragment. The spike-cocktail was diluted to approximately 120,000 copy number per microlitre for experiments. (A separate spike-cocktail was constructed that contained unmethylated spikes to assess aspects of specificity.)

#### 2.2.3 Endogenous human genomic DNA

**Fig. 1:**
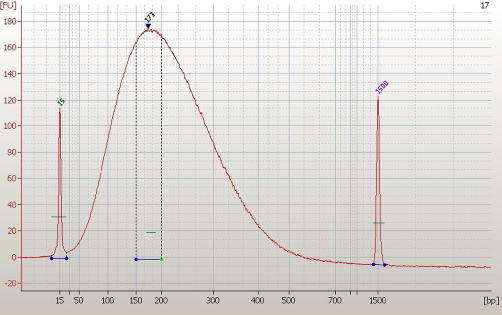
Sonicated DNA. An electropherogram produced using an Agilent Bioanalyzer 2100 illustrating the size-range of DNA used in AutoMeDIP-seq. Approximately, 45% of the sample is contained between 150 and 250 bp.

Human genomic DNA was used as an endogenous reference to maintain reaction stoichiometry. Whole blood was extracted and pooled from 10 human individuals. DNA was purified in 10 ml batches using Invitrogen GeneCatcher kits according to the manufacturer’s instructions. All purified batches were re-pooled, quantified using spectrophotometry and run on 2% agarose gels to confirm high (>10 Kb) molecular weight. Pooled-blood DNA was diluted to 100 ng/μl and sonicated in 10 μg batches using 1.5 ml tubes. The Bioruptor was set to ‘high’ (320 W) for ten 15-min cycles, each consisting of 30 s ON/30 s OFF. After each cycle the sonicator tank was replenished with ice-cold (but ice-free) water. (We have found the presence of ice in the tank contributes to the accumulation of droplets on the tube-wall and lid, which then avoid sonication.) The electropherogram in Fig. 1 illustrates the size-range of DNA fragments after sonication; we aim to obtain >20% of the sample between 150 and 200 bp, and more than 90% <500 bp.

We recommend starting MeDIP-seq with 6 μg genomic DNA. This amount takes into account losses associated with purification steps between Illumina GAII library preparation, and the iterative nature of verifying sonicated fragments (we have found DNA from different sources also influences sonication time).

### 2.3 Automation

#### 2.3.1 Diagenode SX-8G IP-Star

**Fig. 2:**
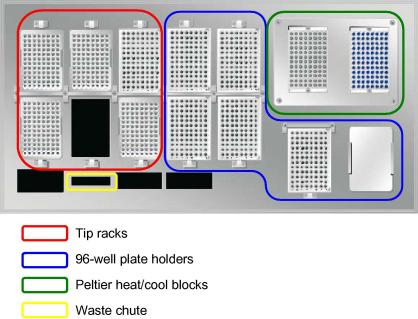
IP-Star bed. The IP-Star contains multiple locations for tips and plates, which will provide increased flexibility for additional automated protocols.

The SX-8G IP-Star (referred to henceforth as “IP-Star”) is a bench-mountable robotic platform. It weighs 130 kg and has a footprint of 1070 mm (*W*) × 650 (*D*) × 770 (*H*). Additional headroom (470 mm) is also required to open the fascia and gain access to the bed of the IP-Star (see Fig. 2). The bed consists of two 96-well Peltier heat blocks compatible with 96-well skirtless plates; six modules to hold 96-well skirted plates at room temperature; and six 96-tip-holder modules. A single 8-channel head uses 200 μl disposable filter tips to move about *x*-, *y*- and *z*-axes to aspirate, dispense and mix liquid. Dispensing-volume tolerances are estimated at <20%, <5% and <2% for dispensations between 5–9, 10–19 and 20–200 μl, respectively. The IP-Star executes automated protocols and individual movements via a graphical user interface (GUI) that currently runs on Windows XP operating systems.

Depending on the number of IPs per run, MeDIP is performed on either one (1–8 MeDIPs) or both (9–16 MeDIPs) Peltier heat blocks. A single MeDIP is currently performed in a 12-strip tube. Each DNA sample requires a single 12-well row of the heat block, and thus, a single channel of the 8-channel dispensing head.

Reagents, sonicated DNA sample, and λ-DNA spike-ins are dispensed into a 12-strip tube prior to starting an AutoMeDIP. AutoMeDIP is started via the GUI and the IP-Star moves sequentially across the wells performing the first three (of four) MeDIP stages: (1) bead washing, (2) immunoselection, and (3) immunoprecipitation. At stage (4) (methylated DNA isolation) a user intervention step is required to dispense Input DNA into one well 1, and proteinase K into wells containing immunoprecipitated DNA (well 12) and newly added Input DNA (well 1).

### 2.4 Workflow

**Fig. 3:**
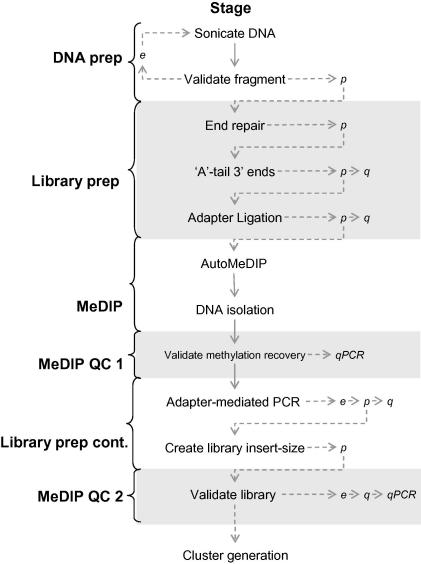
AutoMeDIP-seq workflow. It is feasible to process up to 16 samples simultaneously in 2 days including analysis of the two QC steps. When processing more than 16 samples it is recommended that each stage (within curly braces) is completed for all samples before moving onto the next stage. e, electrophorese, p, purify, q, quantitate, qPCR, quantitative polymerase chain reaction.

The AutoMeDIP-seq workflow is presented in Fig. 3. It is our preference to perform MeDIP between steps 3 and 4 of Illumina GAII library prep to avoid ligating adapters to λ-DNA spike-ins (for MeDIP QC only), and thus circumvent their sequencing during cluster generation. We also recommend running a ‘reference’ DNA sample alongside study samples for quality control purposes toward the end of the method. Where appropriate, this sample is referred to as the ‘reference’ DNA sample.

#### 2.4.1 Illumina GAII Paired End sample prep (steps 1–3 of 5)

Purify the sonicated DNA on QIAGEN QIAquick columns/plates according to the manufacturer’s instructions and elute in 31 μl buffer EB (recovered volume is 30 μl). Quantitate the DNA using spectrophotometry.

Step 1 is an end-repair reaction. In a 100 μl final reaction volume, add 45 μl H_2_O, 10 μl T4 DNA ligase buffer (with 10 mM ATP), 4 μl dNTP mix, 5 μl T4 DNA polymerase, 1 μl Klenow DNA polymerase, and 5 μl T4 Polynucleotide kinase (PNK) to the 30 μl genomic DNA; incubate at 20 °C for 30 min then purify on QIAGEN QIAquick columns/plates and elute in 33 μl buffer EB.

Step 2 creates an A base overhang to the 3′ ends of end-repaired fragments to prepare them for adapter-ligation. In a final volume of 50 μl, add 5 μl Klenow buffer, 10 μl dATP, and 3 μl Klenow fragment (3′–5′ exo^−^) to the 32 μl DNA sample; incubate at 37 °C for 30 min then purify on QIAGEN MinElute columns/plates and elute in 10 μl buffer EB. Quantitate the DNA.

Step 3 (final step before MeDIP) ligates adapters to the ends of the DNA fragments to facilitate adapter-mediated enrichment by PCR prior to cluster generation. This step is optimised for 5 μg starting template so the proportion of adapters added should be titrated accordingly and the reaction volume made up with buffer EB. On the basis of 5 μg starting template, add 25 μl DNA ligase buffer, 10 μl adapter oligo mix, and 5 μl T4 DNA ligase to the 10 μl sample DNA to create a 50 μl final reaction volume; incubate at 20 °C for 15 min then purify on QIAGEN QIAquick columns/plates and elute in 30 μl buffer EB.

Measure the DNA concentration following purification: the yield of DNA should have increased due to incorporation of ∼65 bp of adapter sequences at fragment ends. The concentration should therefore be adjusted to maintain the appropriate molar ratio between genomic DNA and anti-5-methylcytidine during MeDIP. This is accomplished by dividing the observed spectrophotometric read (*conc_obs_*) by the ratio of post-adapter-ligated fragment size to average pre-adapter-ligated fragment size to give a concentration of genomic DNA (*conc_genomic_*):

(1) $${\mathrm{conc}}_{\mathrm{genomic}}=\frac{{\mathrm{conc}}_{\mathrm{obs}}}{\left\{\frac{{\overset{¯}{\mathrm{bp}}}_{\mathrm{sample}}+\sum {\mathrm{bp}}_{\mathrm{adapters}}}{{\overset{¯}{\mathrm{bp}}}_{\mathrm{sample}}}\right\}}$$

rearranged to give:

(2) $${\mathrm{conc}}_{\mathrm{genomic}}=\frac{{\mathrm{conc}}_{\mathrm{obs}}\times {\overset{¯}{\mathrm{bp}}}_{\mathrm{sample}}}{{\overset{¯}{\mathrm{bp}}}_{\mathrm{sample}}+\sum {\mathrm{bp}}_{\mathrm{adapters}}}$$

where ${\overset{¯}{\mathrm{bp}}}_{\mathrm{sample}}$ is the average size (bp) of the sonicated genomic DNA sample, and $\sum {\mathrm{bp}}_{\mathrm{adapters}}$ is the sum of adapter lengths (bp) in the adapter mix. For example, a spectrophotometric read of 100 ng/μl for a sample with an average pre-adapter-ligated fragment size 175 bp, would yield *conc_genomic_* of 72.9 ng/μl. Given the 30 μl eluate, this equates to approximately 2.2 μg of DNA sample suitable for MeDIP.

#### 2.4.2 AutoMeDIP using the IP-Star

**Fig. 4:**
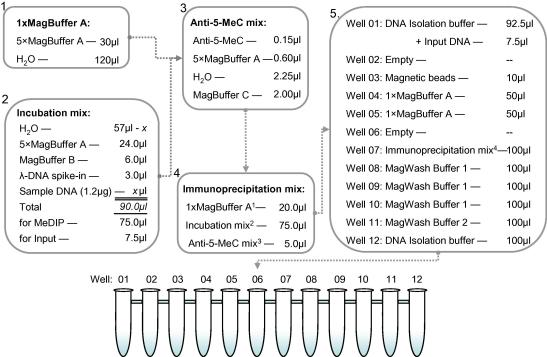
Reaction mixes for a single AutoMeDIP run. Reagents are mixed and dispensed into 12-strip-tubes prior to starting an AutoMeDIP run, at which point the IP-Star performs MeDIP-sequentially across the wells. Prior to dispensing the incubation mix, DNA must be denatured, otherwise the MeDIP reaction is ineffective.

Reagents and reaction mixes are dispensed by hand prior to starting an AutoMeDIP run and are illustrated in Fig. 4. Because MeDIP only works using single-stranded DNA, the first reaction mix (“incubation mix”) needs to be denatured. To prepare the incubation mix, add 1.2 μg adapter-ligated DNA, 24 μl 5× MagBuffer A, 6 μl MagBuffer B, 3 μl λ-DNA spike-in (diluted to contain ∼120,000 copies per μl), and make up to 90 μl with H_2_O and pipette mix; incubate at 100 °C for 10 min, spin briefly if necessary, and snap-cool for 10 min on ice. The incubation mix is prepared 20% in excess; 75 μl is used for MeDIP and 7.5 μl is reserved for Input. Input DNA is not usually sequenced but used for quality control purposes (see Section 3.2).

The second reaction mix – “antibody mix” – is prepared on ice: in the following order add 2 μl MagBuffer C, 0.6 μl 5× MagBuffer A, 0.15 μl anti-5-methylcytidine, and 2.25 μl H_2_O and pipette mix. Because antibody is supplied at 1 μg/μl, 150 ng anti-5-methylcytidine is used per MeDIP – a CpG:anti-5mC molar ratio of approximately 25:1.

Dispense reagents into their appropriate wells as indicated in Fig. 4. Use inverse pipetting when dispensing reaction mixes to avoid bubbles and the introduction of dead volumes; because the IP-Star mixes during the automated protocol it is not necessary to pipette mix when dispensing single reagents.

Switch on the IP-Star and controlling computer. Open the GUI and the IP-Star door. When performing less than eight MeDIPs, place six tips per row in a tip-holder module and place strip-tubes containing the reagents and reaction mixes in the corresponding rows on the right-hand Peltier heat block; when performing between 9 and 16 MeDIPs, place nine tips per row in a tip-holder module and place strip-tubes in corresponding rows across both Peltier heat blocks. Close the door of the IP-Star. Double-click the graphical representation of appropriate tip-holder module until a full module of tips appears. Open the Protocol folder, select the relevant protocol and click “Start”. Alternatively, click “Modify” to specify the immunoselection time and temperature; changes can be made, saved, and then executed via the screens that follow. (Our preference is at least 7.5 h immunoselection at 4 °C.) A final confirmation window will appear; click “OK” to execute the chosen automated protocol or “CANCEL” to abort. Upon clicking “OK” the automated protocol will commence as previously described (see Section 2.3).

After the final immunoprecipitation in MagWash Buffer 2, the methylated DNA/anti-5mC/bead complex is eluted in 100 μl DNA Isolation Buffer. Follow the instruction in the dialogue box that appears: for each MeDIP add 1 μl proteinase K to wells 1 and 12, and 7.5 μl incubation mix (containing Input DNA) into well 1. Pipette mix the contents of well 12 after adding proteinase K to ensure the beads are in suspension. Cap the column of wells 1 and 12 using 8-strip tube caps. Click “OK”. Follow the instructions of the second dialogue box: close the IP-Star door. Click “OK”. The IP-Star will activate the enzyme by heating to 55 °C for 15 min, then denature the enzyme by heating to 95 °C for a further 15 min, then finally cool the sample to 4 °C where the IP-Star holds this temperature until switched off. The AutoMeDIP reaction is complete.

#### 2.4.3 Capture the methylation-enriched fraction of DNA

Upon completion of the run, open the door and remove all strip-tubes from the Peltier blocks. If no further reactions are to be performed, exit the GUI and turn off the IP-Star. Separate the wells containing Input DNA (well 1) and MeDIP DNA (well 12) from the rest of the strip-tubes and briefly spin in a microfuge to collect any condensate. Place the tubes in a magnetic rack and leave for 10 min in a fridge or on ice to capture the beads. Remove the supernatant of both Input and MeDIP and place in clean, labelled tubes. Discard the beads. Proceed to QC.

#### 2.4.4 MeDIP QC 1

The first MeDIP quality control directly assesses the efficiency of the MeDIP reaction using quantitative PCR (qPCR). Because qPCR efficiency improves with shorter fragments we designed nested qPCR primers within each fragment (see Table 1). qPCRs for each region are performed in triplicate for both Input and MeDIP DNA. Per qPCR assay add 6.25 μl 2× SYBR reaction buffer (including 8 mM MgCl_2_), 0.625 μl primer pair (10 μM forward and 10 μM reverse), 4.375 μl H_2_O, and 1.25 μl DNA sample. Run with the following thermal cycling conditions: (a) 95 °C for 5 min, (b) 40 cycles of 95 °C for 15 s, 60 °C for 15 s (including single fluorescence read per cycle), (c) dissociation curve.

MeDIP efficiency is indexed by recovery, which is calculated using the cycle threshold (*C_t_*) values of MeDIP ($C_{t_{\mathrm{MeDIP}}}$) and Input ($C_{t_{\mathrm{Input}}}$) samples. Because the proportion of Input DNA in 100 μl DIB solution is 10% of that used at the start of MeDIP, Input *C_t_* values are adjusted to represent 100% starting template by subtracting a number of Cts ($C_{t_{\mathrm{Input}\_\mathrm{adj}}}$):

(3) $$C_{t_{\mathrm{Input}\_\mathrm{adj}}}=\frac{\log({\%}_{\mathrm{Input}})}{\log(2\mathrm{AE})}$$

where %*_Input_* is the proportion of Input (to MeDIP) used and *AE* is the amplification efficiency of the primers (expressed as a decimal and determined empirically). Recovery is calculated as:

(4) $$\mathrm{recovery}(\%)=2{\mathrm{AE}}^{\left((C_{t_{\mathrm{Input}}}-C_{t_{\mathrm{Input}\_\mathrm{adj}}})-C_{t_{\mathrm{MeDIP}}}\right)}\times 100$$

Incorporating recovery of unmethylated spike-ins allows the calculation of specificity. (For the purposes of this paper we conducted separate experiments containing an unmethylated spike-cocktail; see Section 3 for how we implement unmethylated and methylated spike-ins within the same DNA sample for QC.) Specificity is calculated as:

(5) $$\mathrm{specificity}=1-\left\{\frac{{\mathrm{recovery}}_{\mathrm{unmeth}}}{{\mathrm{recovery}}_{\mathrm{meth}}}\right\}$$

Although this can be more directly expressed in terms of Cts:

(6) $$\mathrm{specificity}=1-(C_{t_{\mathrm{meth}}}-C_{t_{\mathrm{unmeth}}})$$

it is more convenient to use Eq. (5) in a study setting, as methylated and unmethylated spike-ins are genomically distinct regions, which have their own amplification efficiencies that can be accounted for (see Section 3).

#### 2.4.5 Illumina GAII sample prep parts 4–5

Step 4 of library prep is an adapter-mediated PCR to enrich the presence of methylated fragments for hybridisation to the flowcell and cluster generation. In a final reaction volume of 50 μl, add 25 μl Phusion High-Fidelity DNA Polymerase, 1 μl PE Primer 1.0, and 1 μl PE Primer 2.0, to 23 μl MeDIP DNA (DNA does not need to be purified). For final QC purposes (see Section 2.4.6), setup a separate PCR to also amplify the Input fraction of ‘reference’ DNA. Incubate on a thermal cycler with the following conditions: (a) 98 °C for 30 s, (b) 12 cycles of 98 °C for 10 s, 65 °C for 30 s, and 72 °C for 30 s, (c) 72 °C for 5 min, and (d) 4 °C hold. Electrophorese 5 μl PCR product on a 2% agarose TBE gel (or Bioanalyzer) to confirm the PCR has worked: a smear (shifted ∼120 bp higher than the pre-library prep sonicated DNA) should be visible. Purify on QIAGEN QIAquick columns/plates, elute in 30 μl buffer EB and quantitate.

Step 5 of sample prep creates a library insert size for cluster generation. To avoid cross-contamination of samples, prepare separate 2% gels for each MeDIP sample using 100 ml 1× TAE, 2 g low melting-point agarose, 50 μg ethidium bromide (final EtBr concentration: 0.5 μg/ml), and 6 mm-wide gel combs. After mixing each sample with an appropriate quantity of loading dye, dispense the sample between two lanes of 50 bp ladder, with at least 2 cm between each loaded lane. Electrophorese for 60 min at 120 V. To prevent cross-linking the DNA, place a strip of aluminium foil on a UV transilluminator, sufficient to cover the lane of MeDIP sample. Because the gel is fragile, place it on the UV transilluminator with care so that the MeDIP sample lane is on top of the foil. Using a UV face shield, switch on the UV transilluminator. (Because of the foil, only the 50 bp ladder should be visible.) With a clean scalpel and ruler, cut two vertical lines either side of the MeDIP sample lane; using same scalpel, make a horizontal cut at 300 bp; with a clean scalpel, make a horizontal cut at 350 bp; very carefully excise the 50 bp range and place in a 1.5 ml tube. Two points are worthy of note here: firstly, the sample size-range has increased ∼120 bp due to the incorporation of PCR primer sequences. Therefore, by excising 300–350 bp this corresponds to selecting ∼180–230 bp of the original genomic DNA size-range. Secondly, although we choose to sequence this size-range, we recommend excising other 50 bp ranges across the full sample size-range (i.e., 220–620 bp) for contingency purposes.

Repeat the excision process for all MeDIPs and the ‘reference’ Input sample. Purify each sample using a QIAGEN Gel extraction kit according to the manufacturer’s instructions, with the exception of dissolving agarose at room temperature (see [12]) and elute in 30 μl. Proceed to MeDIP QC 2.

#### 2.4.6 MeDIP QC 2

The purpose of the second QC is to verify the library insert remains enriched for methylated fragments and depleted for unmethylated fragments following PCR and gel excision. Because the λ-DNA fragments are added after adapter-ligation, they are immune to amplification during PCR and thus diluted-out of the MeDIP. Therefore, MeDIP QC 2 tests rely on the endogenous genomic DNA.

Two regions likely to be differentially methylated in humans, and the primers to assay these regions, are presented in Table 1. Although consistent methylation states of these regions have been shown previously in 13 normal somatic tissues, placenta, sperm, and an immortalized cell line [10], we recommend ascertaining the methylation state of your ‘reference’ sample *a priori*: a simple MeDIP reaction is often sufficient.

Following purification, quantitate all MeDIP samples and the ‘reference’ Input sample by spectrophotometry. The DNA yield should be between 60 and 500 ng and the 260/280 ratio should be >1.8. Normalize the concentrations of ‘reference’ MeDIP and Input (e.g., 1 ng/μl) and set up triplicate qPCRs for the two genomic regions using the reaction mix and thermal cycling conditions described above (see MeDIP QC 1). Calculate the fold-enrichment ratio for methylated vs. unmethylated regions:

(7) $$\text{fold-enrichment ratio}=\frac{2{\mathrm{AE}}^{\left(C_{t_{\mathrm{Input}\_\mathrm{meth}}}-C_{t_{\mathrm{MeDIP}\_\mathrm{meth}}}\right)}}{2{\mathrm{AE}}^{\left(C_{t_{\mathrm{Input}\_\mathrm{unmeth}}}-C_{t_{\mathrm{MeDIP}\_\mathrm{unmeth}}}\right)}}$$

In the presence of equally efficient primers, the fold-enrichment ratio can be calculated without Input DNA (i.e., $2{\mathrm{AE}}^{\left(C_{t_{\mathrm{MeDIP}\_\mathrm{unmeth}}}-C_{t_{\mathrm{MeDIP}\_\mathrm{meth}}}\right)}$; however, we recommend running at least one Input to verify each primer. The pass threshold is discussed in Section 3.2.

As a final validation, run 1 μl sample on an Agilent Bioanalyzer to check the size-range corresponds to that which was excised. Following successful QC, quantitate the sample using either fluorometry or a TaqMan assay (see [12] for details) to create a 10 nM dilution used for cluster generation. The methods involved with cluster generation are often handled independently by dedicated personnel at the sequencing institute; for this reason, this paper does not detail these methods.

## 3 Results and discussion

Recent sequencing efforts have revealed the confluence of rare genetic alterations on phenotypic plasticity. DNA methylation is no exception and analyses need to encompass the whole genome. Moreover, given the individual-specific nature of these events, large cohorts are required to capture the rare but crucial alterations. As a step toward achieving these goals we present AutoMeDIP-seq, a whole genome sequencing technique incorporating an automated MeDIP step that offers increased reproducibility, accuracy and throughput for assessing the methylated fraction of a genome (the “methylome”).

### 3.1 Performance

**Fig. 5:**
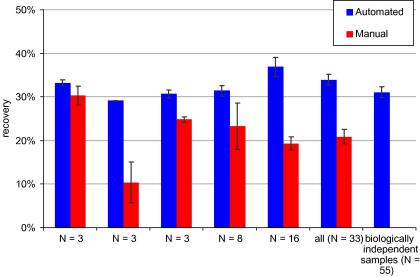
Reproducibility indexed as a function of recovery for a spiked-in methylated fragment. All but the last category show reproducibility of a methylated fragment (20CpGs) spiked-in against identical human genomic DNA (pooled-blood DNA); the last category involves MeDIPs performed using 55 independent DNAs from an empirical study. The first five categories represent *N* simultaneous MeDIPs performed on separate days and the penultimate category summarises these MeDIPs. Error bars are ±1 standard error of the mean. We observed a similar pattern of reproducibility for other fragments (data not shown).

A key advantage of automated protocols over manual protocols is increased reproducibility, and the IP-Star is no exception. Fig. 5 illustrates reproducibility (as a function of “20CpG” recovery; other fragments not shown) for a total of 33 MeDIPs performed on five consecutive days. By way of comparison we also performed MeDIPs using an established manual protocol performed in 1.5 ml microcentrifuge tubes (see [10]). As Fig. 5 shows, the automated protocol shows a tighter range of recovery, both within- and between-days compared with the manual protocol. Moreover, recovery is marginally (but consistently) higher for the automated protocol than the manual protocol, although we accept that different immunocapture incubation periods for each protocol were used (manual: 4 h vs. automated: 7.5 h).

**Fig. 6:**
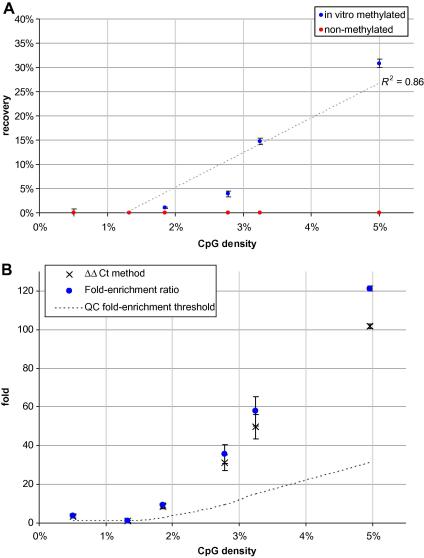
Recovery and relative enrichment as a function of CpG density. (A) Recovery of methylated fragments showed a clear positive correlation with CpG density, while non-methylated fragments were consistently unrecovered (<1%), regardless of CpG density. (B) Following adapter-mediated PCR, MeDIP and Input sample concentrations were equalized; we calculated fold-enrichment ratios (blue spots) and – for comparison – relative enrichment using the ΔΔ*C_t_* method (black crosses), by normalizing the Δ*C_t_* for each methylated fragment against the average Δ*C_t_* for all unmethylated fragments. The dashed line indicates a suggested fold-enrichment threshold for quality control purposes and is contingent on the CpG density of the region tested; after gel-purification, samples that pass this QC threshold and also match the expected size-range are suitable for cluster generation. Error bars are ±1 standard error of the mean using three independent AutoMeDIPs.

Fig. 6A demonstrates that recovery of fragments is positively correlated with CpG density (blue spots). It also shows that this relationship is both strong and that the majority of the variance is explained linearly (*R*^2^ = .86), which is reassuring when compared to previous analogous experiments (e.g., [13]). Following adapter-mediated PCR we normalized all MeDIP and Input DNA concentrations to 1 ng/μl, so as to calculate fold-enrichment ratios (described in Section 2.4.6) for each methylated fragment. For comparison we also calculated fold-enrichment scores using the traditional ΔΔ*C_t_* method [14]. Readers should be aware that because λ-DNA spike-ins are added at the point of setting up AutoMeDIP, they avoid amplification via adapter-mediated PCR; however, because we want to show the effect of adapter-medicated PCR on the fragments, for the purposes of this paper we conducted an experiment by adding λ-DNA spike-ins to the sonicated genomic DNA at the point of starting end-repair. In both calculations we anchored the observed Δ*C_t_* of each of the six methylated fragments to the average Δ*C_t_* of all six unmethylated fragments (average $C_{t_{\mathrm{unmethInput}}}-C_{t_{\mathrm{unmethMeDIP}}}$ = −4.4). The resulting scores are plotted in Fig. 6B, which illustrates that the original recovery profile is largely maintained after the samples have been amplified by adapter-mediated PCR (*R*^2^ = .92). Similar to data shown in Fig. 5, reproducibility is high across all the six regions as indexed by the small error bars (±1 standard error) at each point. Although regions of very low CpG density (CpG% <1.9) are not greatly enriched, MeDIP is capable of enriching not just CpG islands, but also CpG island shores, which is important considering their importance as DMRs in phenotypic plasticity [3,15]. We note however, that generating enough reads to cover regions of very low CpG density requires increased sequencing depth, which significantly impacts on cost.

To assess AutoMeDIP specificity, and show recovery of methylated fragments is not random, an unmethylated spike-cocktail was run in parallel to the *in vitro* methylated version. Fig. 6 shows that unmethylated fragments (red spots) were routinely not recovered (range: 0.00–0.15%) and CpG density is not a factor, as expected. Just focussing on discernibly recovered fragments (i.e., CpG% ⩾2.0), these recovery rates translate to specificity estimates ranging from 96.3% (CpG% = 2.8) to 99.9% (CpG% = 5.0).

### 3.2 QC considerations

As alluded to in the Methods, implementing these metrics in a substantive study requires additional consideration. The specificity metrics presented here refer to the ratio of methylated-to-unmethylated recovery of the *same* fragment in two *separate* assays; to quality control assays in a substantive study we need to assess the ratio of methylated-to-unmethylated recovery of *different* fragments within one *discrete* assay. To remedy this we spike-in equimolar amounts of unmethylated “15CpG” and methylated “20CpG”. In doing so however, we need to bear in mind that recovery varies by CpG density, and specificity will be inflated if more efficiently-recovered fragments are used as methylated controls.

To define reasonable criteria for a QC specificity threshold we draw on previous experience. As shown earlier, recovery of methylated “20CpG” is consistent and reliable when spiked-in against our pooled-blood DNA. However, spiking-in against different study samples (i.e., different methylation profiles) alters reaction stoichiometry and thus recovery; in biologically independent samples tested to date, we have found recovery of methylated “20CpG” ranges from 4.6% to 54.4% (mean = 30.8%, SD = 9.2%) and recovery of unmethylated “15CpG” ranges from 0.01% to 0.87% (mean = 0.11%, SD = 0.20%). Therefore, the first step in defining a suitable QC specificity threshold is to define baseline recovery estimates: we ranked our previous recovery rates of methylated “20CpG” and unmethylated “15CpG” in order of “ideal recovery” (e.g., 1 = high recovery of methylated fragment OR low recovery of unmethylated fragment); we then selected recoveries corresponding to the 90th percentiles (methylated fragment = 21.5%, unmethylated fragment = 0.39%) and used Eq. (5) to generate a specificity threshold of 98.2% (or approximately 50-fold difference in recovery of methylated fragments over unmethylated fragments). We feel this current threshold provides a reasonable cut-off for quantitatively removing outliers/failures: in our most recent study, 51 of 52 samples passed this QC threshold and were taken forward for sequencing. The mean specificity of samples in this study was 99.6% (SD = 0.7%), which translates to ∼250-fold recovery difference of methylated vs. unmethylated fragments.

As with MeDIP QC 1, imposing a threshold for MeDIP QC 2 requires consideration of CpG density and therefore needs to be flexible (see Fig. 6B). Focusing on a region of 5% CpG density, MeDIPs should be considered suitable for cluster generation if the fold-enrichment ratio is >∼30, indicating a 30-fold increase of methylated fragments over unmethylated fragments (cf. 50-fold in MeDIP QC 1). The rationale for imposing a less stringent threshold at this stage is due to anticipated loss (∼65%) of methylated DNA during PCR- and gel-purification when compared with the unchanging quantity of unmethylated DNA. In other words, qPCR curves for methylated DNA shift right, whereas qPCR curves for unmethylated DNA remain unchanged. Nevertheless, we routinely see fold-enrichment ratios greater than 100 using primers for endogenous DNA (see Table 1).

### 3.3 Logistics

To process the four key stages of MeDIP for 16 samples ready for the first round of QC, we found the automated protocol cut hands-on time by approximately two-thirds compared with manual methods (40 min cf. 2 h). The automated protocol also has the advantage that hands-on work is spent virtually entirely prior to the MeDIP reactions, meaning users’ time can be split more effectively over other tasks. Most of the hands-on time is saved during bead washing steps, and the fact that post-MeDIP DNA does not need to be purified prior to PCR applications.

In terms of throughput, we currently and routinely perform 16 MeDIPs in 24 h, including initial QC. It may be possible however to double throughput by halving the immunoselection incubation time and increasing the temperature; this has not been tested extensively however, and requires further validation.

## 4 Concluding remarks

The regulatory effects of DNA methylation at single loci are likely to act synergistically with other local patterns of DNA methylation, other epigenetic marks and sequence-based genetic polymorphisms. For this reason, it is desirable for methylation studies to move from a candidate approach to whole genome screens; only in this way will the genomic orchestra be heard, allowing the genetic soloists to take centre stage. Although the gold-standard of methylome assays is MethylC-seq, it remains prohibitively expensive, time consuming and labour intensive. AutoMeDIP-seq offers a reasonable higher-throughput alternative that is reliable, sensitive and specific, albeit without the resolution and genomic coverage of MethylC-seq. One drawback of the method is an obvious enrichment bias toward CpG-dense regions of the genome, however; we show that AutoMeDIP is still capable of reliably enriching for fragments >2%. Moreover, because the reduced read-depth that necessarily follows in these regions can be accounted for *in silico* [7], the methylation state at CpG island shores can still be assessed and incorporated into a hypothesis-free approach to highlight specific regions that can be replicated in alternative – possibly higher resolution – assays.

## Figures and Tables

**Table 1:** PCR primers used to generate λ-DNA fragments and qPCR primers for quantitation of λ-DNA and human DNA regions. The methylated and unmethylated states at human chromosome 22 and 6, respectively were ascertained previously (see [10]).

No. CpGs	CpG (%)	Genome	Protocol	Start	End	Product size (bp)	Primer	Sequence (5′–3′)	Length	GC (%)	*T_A_* (° Fc)
1	0.5	Lambda	PCR	23,967	24,163	197	F	GAGGTGATAAAATTAACTGC	20	35.0	53
							R	GGCTCTACCATATCTCCTA	19	47.4	
			qPCR	24,004	24,148	145	F	ACAAGTTGTTTGATCTTTGC	20	35.0	60
							R	CCTATGAGCAACGTGTTAG	19	47.4	
3	1.3	Lambda	PCR	27,573	27,798	226	F	GCTAATGCTCTGTTACAGGT	20	45.0	53
							R	ACTGATAGTGACCTGTTCGT	20	45.0	
			qPCR	27,629	27,777	149	F	GCATATGTTGTGTTTTACAG	20	35.0	60
							R	GCAACAAATTGATAAGCA	18	33.3	
5	1.9	Lambda	PCR	25,869	26,138	270	F	CATGTCCAGAGCTCATTC	18	50.0	53
							R	GTTTAAAATCACTAGGCGA	19	35.0	
			qPCR	25,942	26,071	130	F	CACTTGAATCTGTGGTTCAT	20	40.0	60
							R	TAGAAAAAGACAACTCTGGC	20	40.0	
10	2.8	Lambda	PCR	33,638	33,997	360	F	CTGACCATTTCCATCATTC	19	42.1	53
							R	GTAACTAAACAGGAGCCG	18	50.0	
			qPCR	33,662	33,786	125	F	GAACTCACACACAACACCA	19	47.4	60
							R	ACTCTGAATACCGACTCAAT	20	40.0	
15	3.2	Lambda	PCR	28,488	28,949	462	F	ATGTATCCATTGAGCATTGCC	21	42.9	53
							R	CACGAATCAGCGGTAAAGGT	20	50.0	
			qPCR	28,669	28,789	121	F	TATCACTGTTGATTCTCGC	19	42.1	60
							R	GGTAAAGAGTTTGGATTAGG	20	40.0	
20	5.0	Lambda	PCR	24,148	24,550	403	F	GAGATATGGTAGAGCCGCAGA	21	52.4	65
							R	CAGTGCCTCGCAAACTCGGAAGA	23	56.5	
			qPCR	24,202	24,309	108	F	GGTGAACTTCCGATAGTG	18	50.0	60
							R	CAGTCATAGATGGTCGGT	18	50.0	
24^a^	4.9^a^	Human	qPCR	Chr22:50216607	Chr22:50216720	114	F	GGGAATATAAGGAGCGCACA	18	55.6	60
							R	TCGGTTAAAACGGTCAGGTC	18	55.6	
25^a^	4.9^a^	Human	qPCR	Chr6:99841600	Chr6:99841709	110	F	CGAGGCGTGAGTTATTCCTG	18	61.1	60
							R	CTCTTGTGGCTGAGCTCCTT	18	61.1	

a CpG count and CpG% estimated by simulation of amplicon-capturing fragments drawn from a random normal distribution.
